# Rational Combination of π‐Conjugated and Non‐π‐Conjugated Groups Achieving Strong Nonlinear Optical Response, Large Optical Anisotropy, and UV Light‐Switchable Fluorescence

**DOI:** 10.1002/advs.202401325

**Published:** 2024-03-13

**Authors:** Danyang Dou, Qi Shi, Huimin Li, Bingbing Zhang, Daqing Yang, Ying Wang

**Affiliations:** ^1^ Hebei Research Center of the Basic Discipline of Synthetic Chemistry Key Laboratory of Analytical Science and Technology of Hebei Province College of Chemistry and Materials Science Hebei University Baoding 071002 China; ^2^ Institute of Life Science and Green Development Hebei University Baoding 071002 China

**Keywords:** π‐conjugation, birefringence, fluorescence, nonlinear optical, optical anisotropy

## Abstract

Combining π‐conjugated and non‐π‐conjugated groups is an important strategy for synthesizing new nonlinear optical (NLO) crystals. However, the second harmonic generation (SHG) response and optical anisotropy can be limited by improper spatial alignment of these functional groups in the crystal structure. In this work, it is revealed that non‐π‐conjugated [NH_2_SO_3_] group acts as both hydrogen bond donor and acceptor, effectively regulating the 2D planar structure formed by π‐conjugated [C_4_N_3_H_6_] groups. The resulting organic–inorganic hybrid crystal C_4_N_3_H_6_SO_3_NH_2_ exhibits a strong SHG response (2.5 × KDP), large optical anisotropy (0.233@546 nm), and blue‐violet and green fluorescence near 360 and 520 nm, respectively. This work expands the methodology for creating new NLO crystals through organic–inorganic hybridization, while also showcasing the potential of C_4_N_3_H_6_SO_3_NH_2_ as a multifunctional optical material.

## Graphical abstract

A multifunctional material C_4_N_3_H_6_SO_3_NH_2_ is synthesized with excellent linear and nonlinear optical properties as well as ultraviolet (UV) light‐switchable fluorescence.

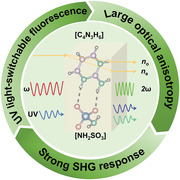

## 1 Introduction

Laser technology is the frontier of science and the strategic support technology with important applications in basic science, information, medical treatment, national defense, and other fields.^[^ ^1^ ^]^ NLO crystal materials greatly expand the wavelength range of laser radiation.^[^ ^2^ ^]^ Ultraviolet (UV) NLO crystal materials with excellent performance need to meet a number of demanding conditions: 1) High transparency in the UV region (*λ*_cut‐off_ < 400 nm); 2) Larger second harmonic generation (SHG); 3) Sufficient birefringence (Δ*n*) for phase matching (PM).^[^ ^3^ ^]^

Based on long‐term exploration, many excellent organic and inorganic π‐conjugated NLO active units, i.e., [BO_3_],^[^ ^4^ ^]^ [B_3_O_6_],^[^ ^5^ ^]^ [NO_3_],^[^ ^6^ ^]^ [CO_3_],^[^ ^7^ ^]^ [C_6_N_7_O_3_],^[^ ^8^ ^]^ [H*_x_* C_3_N_3_O_3_] (*x* = 1–3),^[^ ^9^ ^]^ [C_3_N_3_S_3_],^[^ ^10^ ^]^ [C_3_H_7_N_6_],^[^ ^11^ ^]^ and [C_4_N_3_H_6_]^[^ ^12^ ^]^ have been widely used in designing synthetic NLO materials. These π‐conjugated groups have detached domain π‐orbitals that exhibit large hyperpolarizabilities along the structural planes, giving rise to a large SHG response. Recently, some novel NLO crystals containing π‐conjugated NLO‐active groups have been widely reported, such as A(3‐C_5_H_4_NSO_3_)· *x*H_2_O (A = Li, Ag, K, Rb, Cs, and NH_4_; *x* = 0 and 1),^[^ ^13^ ^]^ ABCO_3_F (A = K, Rb, B = Mg, Ca, Sr),^[^ ^14^ ^]^ Na_10_Zn(NO_3_)_4_(SO_3_S)_4_,^[^ ^6b^ ^]^ K_3_C_6_N_7_O_3_·2H_2_O,^[^ ^8a^ ^]^ KLi(HC_3_N_3_O_3_)·2H_2_O,^[^ ^15^ ^]^ Pb_2_Ba_3_(BO_3_)_3_Cl,^[^ ^16^ ^]^ K_4_(HC_3_N_3_S_3_)_2_·H_2_O,^[^ ^17^ ^]^ and (H_7_C_3_N_6_)(H_6_C_3_N_6_)ZnCl_3_.^[^ ^18^ ^]^

Planar π‐conjugated groups are considered to be excellent birefringent active groups due to their tendency to produce large optical anisotropy differences between the in‐plane and perpendicular plane directions. Many excellent birefringent materials based on microscopic functional units with large anisotropy have been widely reported, such as Na_3_Ba_2_(B_3_O_6_)_2_F,^[^ ^5b^ ^]^ C_3_N_6_H_7_SO_3_NH_2_,^[^ ^19^ ^]^ APbBr_4_ (MLA = melamine),^[^ ^11a^ ^]^ and Cs_2_Mg(H_2_C_3_N_3_S_3_)_4_·8H_2_O.^[^ ^10^ ^]^ Also, numerous results show that planar π‐conjugated organic groups can easily produce fluorescence in π → π* and n → π* leaps, which provides new ideas for us to synthesize multifunctional optical materials.^[^ ^8^, ^12^ ^]^ In addition, using π‐conjugated organic groups as functional motifs, luminescent films prepared by dispersing the corresponding salts in polymer matrices can be applied in the fields of sensing, catalysis, and bioimaging.^[^ ^20^ ^]^

Non‐π conjugated systems are also the focus of research in the development of optical materials, given the requirement for short absorption edges. Conventional tetrahedral groups such as BO_4_, SO_4_, and PO_4_ belong to the *T*_d_ point group, and the high degree of symmetry leads to a very small polarizability dipole. Recently, the exploration of new heteroatom‐substituted tetrahedral groups has opened up new directions for non‐π conjugated systems (i.e., [BO*_x_* F_4−_ *_x_*] (*x* = 1–3),^[^ ^3a^ ^]^ [PO*_x_* F_4−_ *_x_*] (*x* = 1–3),^[^ ^21^ ^]^ and [SiO*_x_* F_6‐_ *_x_*] (*x* = 1–5),^[^ ^22^ ^]^ and a lot of NLO crystal materials with excellent performance have been reported. Surprisingly, the introduction of heteroatoms into the conventional tetrahedral unit leads to a decrease in the symmetry of the units, and the non‐π‐conjugated groups have a stronger hyperpolarizability and greater optical anisotropy, and become new NLO‐active groups. Among heteroatom‐substituted tetrahedral groups, [NH_2_SO_3_] anionic group is unique in one detail of its strong hydrogen bond‐donating and hydrogen bond‐accepting characteristic. The synthesis of a series of excellent NLO crystals and birefringent crystals demonstrates that the [NH_2_SO_3_] group is an excellent functional motif for constructing optical materials.^[^ ^19^, ^23^ ^]^

Organic–inorganic hybrid materials have been proven to exhibit excellent linear optical and NLO performance by combining the benefits of organic and inorganic motifs. Taking the above aspects into consideration, in this work, a multifunctional noncentrosymmetric crystal C_4_N_3_H_6_SO_3_NH_2_ was synthesized by combining planar π‐conjugated [C_4_N_3_H_6_] cations with [NH_2_SO_3_] groups. In addition, single crystal X‐ray diffraction (XRD), powder XRD, energy dispersive X‐ray spectrometry (EDX), infrared (IR) spectroscopy, fluorescence spectroscopy, UV–Vis–NIR diffuse reflectance spectroscopy, thermal analysis, and theoretical calculations were performed for C_4_N_3_H_6_SO_3_NH_2_. Furthermore, we prepared polymer fluorescent films by compounding C_4_N_3_H_6_SO_3_NH_2_ with poly(vinyl alcohol) (PVA), which provides a useful idea for developing environmentally friendly, low‐cost, and flexible luminescent materials.

## 2 Results and Discussion

### 2.1 Crystal Structure

**Figure 1:**
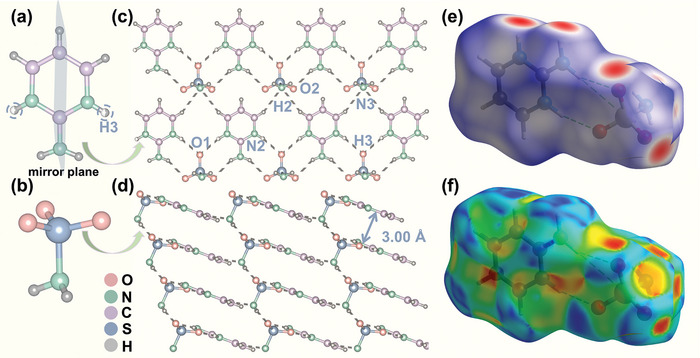
The crystal structure of C_4_N_3_H_6_SO_3_NH_2_. a) The planar [C_4_N_3_H_6_] unit (the occupancy of the circled atoms is 50%); b) The tetrahedral [NH_2_SO_3_] group; c) The 2D planar structure formed by hydrogen bonding of [C_4_N_3_H_6_] and [NH_2_SO_3_] groups on the *bc* plane (d)View of 3D network structure along the *b*‐direction; e) Hirshfeld surfaces plotted over *d*_norm_ and f) shape index for C_4_N_3_H_6_SO_3_NH_2_.

The C_4_N_3_H_6_SO_3_NH_2_ crystallizes in the noncentrosymmetric monoclinic space group of *Cm* (No. 8). The crystal data and structure refinements for C_4_N_3_H_6_SO_3_NH_2_ are listed in Table S1 (Supporting Information). In this crystal structure, the numbers of independent atoms for C, N, H, S, and O atoms are 4, 3, 6, 1, and 2, respectively. The basic structural units in C_4_N_3_H_6_SO_3_NH_2_ are the planar [C_4_N_3_H_6_] cation (**Figure** **1**a) and tetrahedral [NH_2_SO_3_] anionic group (Figure 1b), with the formal charge of +1 and −1, respectively. Noted that the [C_4_N_3_H_6_] cation is located in the mirror plane, and the occupancy of H3 atoms is set as 50% to keep charge balance. Due to the symmetry constraint, there is no deviation of different C─N and C─C bond lengths in [C_4_N_3_H_6_] cation, unlike other 2‐aminopyrimidinium salts.^[^ ^12b^ ^]^ The [C_4_N_3_H_6_] rings are arranged in parallel on the *bc* plane, and all the groups are aligned in the same direction. This planar arrangement of structures makes a positive contribution to macroscopic optical anisotropy. The same is true for the [NH_2_SO_3_] units. All [NH_2_SO_3_] groups are arranged parallel in the *b*‐direction and point in the same direction. In particular, on the *bc* plane, the [C_4_N_3_H_6_] groups are not directly attached, but instead, they are tightly linked through hydrogen bonds formed with the [NH_2_SO_3_] groups (N1─H1⋯O2, N3─H2⋯O2, N2─H3⋯O1, and C2─H4⋯N3) which in turn form the 2D planar structure (Figure 1c). Meanwhile, these hydrogen bonds formed between the anions and cations securely hold the [NH_2_SO_3_] groups in place between the layers. Additionally, in the *b*‐direction, the S─N bonds of the [NH_2_SO_3_] groups act as bridges connecting the 2D planar structure formed by [C_4_N_3_H_6_] with an interlayer distance of 3.00 Å (see Figure 1d). This ultimately results in the formation of a 3D network structure.

The decorated Hirshfeld surface and its corresponding 2D fingerprint^[^ ^24^ ^]^ were generated to visualize quantitative intermolecular interactions and crystal packing mode of C_4_H_6_N_3_SO_3_NH_2_. Figure 1e represents Hirshfeld surfaces plotted over the normalized contact distance (*d*_norm_) and shape index (Figure 1f). The red regions in Hirshfeld surfaces mapped with *d*_norm_ indicate the presence of the N─H⋯O and C─H⋯N interactions in crystal packing, while the triangular‐shaped regions on the shape index surface around π‐aromatic [C_4_N_3_H_6_] rings are diagnostic for π⋯π stacking interactions. It is noted that the π⋯π stacking interaction (including edge‐to‐face C─H⋯π and face‐to‐face π⋯π interactions) is very weak since the centroid‐to‐centroid separation of [C_4_N_3_H_6_] rings is 5.192 Å. Using the decomposed 2D fingerprint plots, the contributions from particular kinds of interactions can be quantified. We can see from Figure S1 (Supporting Information) that O⋯H and N⋯H interactions make up 50.7% of the Hirshfeld surface, while H⋯H and C⋯H interactions contribute up to 31.0% and 11.3%, respectively. The other interactions including C⋯C, C⋯O, and N⋯O have comparatively much less contribution to crystal packing. Clearly, hydrogen bonds, especially the N─H⋯O bonds, dominate the packing motifs of C_4_H_6_N_3_SO_3_NH_2_, whereas the π⋯π stacking can be overlooked. The inorganic [NH_2_SO_3_] units contain terminal O atoms and ─NH_2_ groups act as both hydrogen bond donors and acceptors, playing a prominent role in the crystal structure of C_4_H_6_N_3_SO_3_NH_2_.

### 2.2 Chemical Phase and Thermal Stability

**Figure 2:**
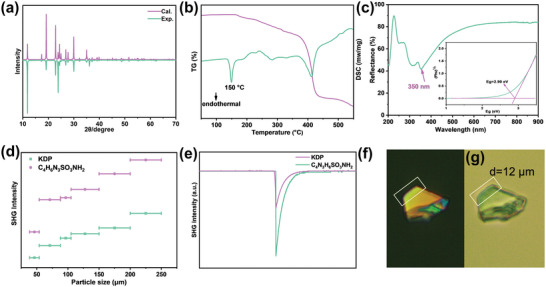
a) The powder XRD; b) TG‐DSC curve; c) UV–vis–NIR diffuse reflectance spectrum; The polycrystalline powder SHG intensity curve d) and SHG response e) at 1064 nm; Crystal picture under polarizing microscope. f) The original color of crystal; g) Crystal achieving complete extinction.

The match between theoretical and experimental XRD patterns proves the purity of the powder, which is the basis for subsequent characterization (**Figure** **2**a). Semiquantitative elemental analysis of the distribution of each element in the crystal of C_4_N_3_H_6_SO_3_NH_2_ was carried out using EDX, and it can be seen that each element distributes in a uniform manner. In addition, the composition of the compound was verified to be correct (Figure S2, Supporting Information). The DSC and TG analysis were conducted to explore its thermal stability. As illustrated in Figure 2b, the DSC curve shows a distinct heat absorption at 150 °C in conjunction with a significant weight reduction observed on the TG curve around this temperature. Thus, the crystals exhibit thermal stability up to 150 °C.

### 2.3 Linear Optical Properties

Characteristic functional groups were identified by analyzing absorption peaks through IR spectroscopy (Figure S3, Supporting Information). For [NH_2_SO_3_] groups and [C_4_N_3_H_6_] groups, *ν*(N─H) at 3320 cm^−1^, 565 cm^−1^, and 1674–1630 cm^−1^, *ν*(N─H⋯O) at 3190 cm^−1^, *ν*(C─H⋯N) at 3090 cm^−1^, *ν*(O─H) at 2790 cm^−1^, ν(P─H) at 2409 cm^−1^, *ν*(C─NH_2_), *δ*(C─NH_2_), and *δ*(N─C─N) at 1480 cm^−1^, *ν*(C─NH_2_), and *δ*(C─H) at 1390 cm^−1^, *β*(NH_2_), and *δ*(C─H) at 1120–990 cm^−1^, *ν*(S─O) at 1230−1050 cm^−1^, *γ*(C─H) at 777 cm^−1^, respectively. Here, *δ*, *ν*, *γ*, and *β* stand for bending vibration, stretching vibrations, and out‐of‐plane and in‐plane bending vibration, respectively. Assignment of these vibration modes verifies the rationality of the structure model.^[^ ^12^, ^19^, ^25^ ^]^ The UV–vis–NIR diffuse reflection spectrum of C_4_N_3_H_6_SO_3_NH_2_ shows that there are several absorptions from 200 to 400 nm, suggesting multiple optical transitions. The UV absorption edge is 350 nm, corresponding to a bandgap of 2.9 eV (Figure 2c). The dense π‐bonding leads to a smaller bandgap as compared to compounds without π‐conjugated groups. Despite this, the results show that C_4_N_3_H_6_SO_3_NH_2_ can be used in near ultraviolet optical range.

### 2.4 NLO Properties

Since C_4_N_3_H_6_SO_3_NH_2_ crystallizes in the NCS space group of *Cm*, its SHG is of interest. The SHG effects of the powder were tested using the modified Kurtz and Perry^[^ ^26^ ^]^ method under coherent optical radiation of 1064 nm generated by a Q‐switched Nd:YAG laser. As shown in Figure 2d, the SHG intensity of the C_4_N_3_H_6_SO_3_NH_2_ compounds increases with increasing particle size, indicating that the compounds can be phase‐matched. The results show that the compounds exhibit a large SHG response of 2.5 × KDP (Figure 2e). The SHG response of C_4_N_3_H_6_SO_3_NH_2_ is comparable to the compounds containing the six‐membered ring (6‐MR) group basic building unit, such as CsAlB_3_O_6_F (2 × KDP),^[^ ^5a^ ^]^ K_2_Pb(H_2_C_3_N_3_O_3_)_4_·(H_2_O)_4_ (2.6 × KDP),^[^ ^9b^ ^]^ and (H_7_C_3_N_6_)(H_6_C_3_N_6_)ZnCl_3_ (2.8 × KDP),^[^ ^18^ ^]^ and greater than that of Cs_3_Na(H_2_C_3_N_3_O_3_)_4_·3H_2_O (2/3 × KDP),^[^ ^27^ ^]^ and (C_3_H_7_N_6_)_6_(H_2_PO_4_)_4_(HPO_4_)·4H_2_O (0.1 × KDP).^[^ ^11b^ ^]^ The large SHG response of C_4_N_3_H_6_SO_3_NH_2_ demonstrated that it is suitable for NLO applications. From a crystal structural point of view, the strong SHG response of C_4_N_3_H_6_SO_3_NH_2_ has a close relation with the coplanar configuration of [C_4_N_3_H_6_] rings, which was evident from other NLO crystals containing π‐conjugated groups.^[^ ^15^, ^28^ ^]^

### 2.5 Experimental Birefringence

The experimental birefringence of C_4_N_3_H_6_SO_3_NH_2_ crystals was measured by the interference color method at 546 nm. using a polarizing microscope equipped with a quartz wedge compensator. The C_4_N_3_H_6_SO_3_NH_2_ crystals used for the birefringence measurements are wedge‐shaped, and therefore the minimum wafer thickness (the edges of the upper half of the crystal, the portion framed by the white lines) was chosen to calculate the birefringence. As shown in Figure 2f, it can be seen that the original interference color of the C_4_N_3_H_6_SO_3_NH_2_ crystal under perpendicular polarized light is six‐order orange (corresponding to an optical range difference of 2800 nm) according to the Michel‐Levy diagram, while the fringes of the upper part of the crystal are completely extinguished after insertion of the compensator (Figure 2g). The thickness of the wedge‐shaped C_4_N_3_H_6_SO_3_NH_2_ wafer is 12 µm as measured by the single crystal diffractometer. Therefore, the birefringence of C_4_N_3_H_6_SO_3_NH_2_ is established to be 0.233 at 546 nm. The experimental birefringence of C_4_N_3_H_6_SO_3_NH_2_ is greater comparable to many compounds containing the 6‐MR group basic building unit, such as KLi(HC_3_N_3_O_3_)·2H_2_O (0.186 @ 514 nm),^[^ ^15^ ^]^ Na_3_Ba_2_(B_3_O_6_)_2_F (0.103 @ 588 nm),^[^ ^5b^ ^]^ CsAlB_3_O_6_F (0.091 @ 1064 nm),^[^ ^5a^ ^]^ K_2_Sr(H_2_C_3_N_3_S_3_)_4_·5H_2_O (0.194 @ 800 nm),^[^ ^10^ ^]^ and K_2_Pb(H_2_C_3_N_3_O_3_)_4_·(H_2_O)_4_ (0.193 @ 532 nm).^[^ ^9b^ ^]^ Therefore, this crystal with large optically anisotropic may have potential application in circulator, optical isolator, Glan‐Taylor prism, and other polarization optic devices.

### 2.6 Fluorescence Properties of C_4_N_3_H_6_SO_3_NH_2_ and C_4_N_3_H_6_SO_3_NH_2_@PVA Films

**Figure 3:**
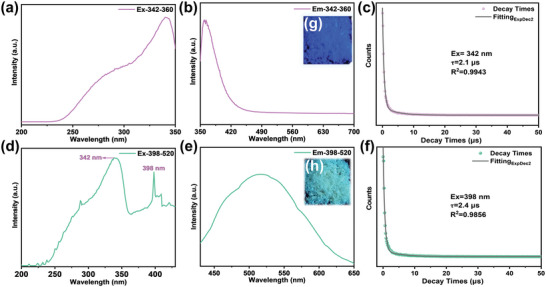
The fluorescence spectra of C_4_N_3_H_6_SO_3_NH_2_; a) The excitation spectrum at 342 nm; b) The emission spectrum at 360 nm; c) The decay curves for C_4_N_3_H_6_SO_3_NH_2_ at 342 nm. d) The excitation spectrum at 398 nm; e) The emission spectrum at 520 nm; f) The decay curves for C_4_N_3_H_6_SO_3_NH_2_ at 398 nm; Digital photos of C_4_N_3_H_6_SO_3_NH_2_ powder under 302 nm g) and 365 nm (h) UV light.

During the experiments, it is interesting to find that the polycrystalline powder samples of C_4_N_3_H_6_SO_3_NH_2_ show tunable colors under UV lamp excitation with different wavelengths. Under the irradiation of a 302 nm UV lamp, the powder exhibited blue‐violet emission color (**Figure** **3**g). Switching the UV lamp wavelength from 302 to 365 nm, the color of powder regulates to blue‐green (Figure 3h). Subsequently, the fluorescence property of C_4_N_3_H_6_SO_3_NH_2_ was investigated. The excitation spectrum of C_4_N_3_H_6_SO_3_NH_2_ in Figure 3a shows a very broad excitation band in the range of 220–350 nm, with a maximum excitation wavelength of 342 nm. Upon excitation at 342 nm, the emission spectrum of C_4_N_3_H_6_SO_3_NH_2_ shows a sharp peak at 360 nm (Figure 3b), which is mainly responsible for the fluorescence emission in blue‐violet. When the excitation wavelength is altered, another powerful excitation peak in the range of 220–400 nm situated at 398 nm can be observed in the excitation spectrum (Figure 3d). The accompanying emission spectrum has a broad emission peak centered at 520 nm (Figure 3e) and was responsible for the blue‐green fluorescence emission. In addition, the 1931 International Commission on Radiation (CIE) chromaticity diagrams obtained from the corresponding emission spectra are shown in Figures S4 (Supporting Information), and the emission color can be easily modulated between blue‐violet and blue‐green by switching the excitation wavelengths between 342 and 398 nm. The UV light‐switchable fluorescence properties of C_4_N_3_H_6_SO_3_NH_2_ can be well understood by considering the π → π* and n → π* electron transitions of [C_4_N_3_H_6_] cation.^[^ ^12b^ ^]^ Furthermore, the corresponding luminescence decay times (τ) of C_4_N_3_H_6_SO_3_NH_2_ excited at 342 and 398 nm were also determined as shown in Figure 3c,f. They follow a double‐exponential function with the values of τ were 2.1 and 2.4 µs Meanwhile, the fluorescence quantum yield excited at 342 nm of the C_4_N_3_H_6_SO_3_NH_2_ powder was evaluated to be 23%.

**Figure 4:**
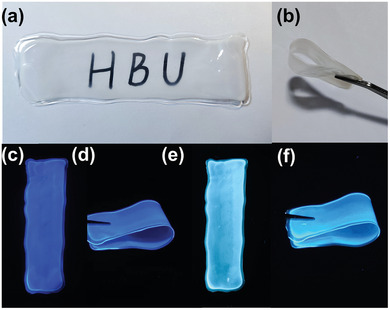
Photographs of C_4_N_3_H_6_SO_3_NH_2_@PVA. a,b) Under daylight; c,d) Under 302 nm UV illumination; e,f) Under 365 nm UV illumination.

Since C_4_N_3_H_6_SO_3_NH_2_ exhibits interesting UV light‐switchable fluorescence, we seek to prepare luminescent films using C_4_N_3_H_6_SO_3_NH_2_ as the building blocks and embed into water soluble PVA, named as C_4_N_3_H_6_SO_3_NH_2_@PVA. The obtained films exhibited good transparency and flexibility (**Figure** **4**a,b). Consistent with the powder samples, the composite films also exhibit UV light‐switchable emission color. They display blue‐violet color when irradiated with a UV lamp at a wavelength of 302 nm, while blue‐green emission is observed under 365 nm irradiation (Figure 4c–f). This flexible and color tunable fluorescent film could have a wide range of applications in collapsible optoelectronic devices, biomaterials, anti‐counterfeiting technology, and smart response.

### 2.7 Theoretical Calculations

**Figure 5:**
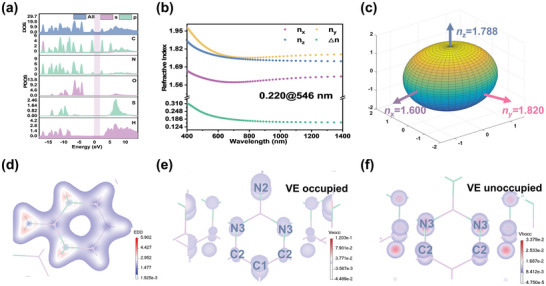
Theoretical calculations of C_4_N_3_H_6_SO_3_NH_2._ a) The DOS and PDOS; b) The calculated birefringence; c) Triaxial ellipsoid of three principal refractive indices at *λ* = 546 nm; d) The EDD of C_4_N_3_H_6_SO_3_NH_2_; e) The VE‐occupied and f) VE‐unoccupied SHG density.

Based on the first principles, the relationship between optical properties and structure has been analyzed using density functional theory within the CASTEP module.^[^ ^29^ ^]^ The calculated bandgap is 1.7 eV by using the HSE06 algorithm^[^ ^30^ ^]^ (Figure S5, Supporting Information). The density of states (DOS) and partial density of states (PDOS) of C_4_N_3_H_6_SO_3_NH_2_ show that both the orbitals in the upper space of the valence band (VB) and the bottom space of the conduction band (CB) are occupied by *C*‐2p and *N*‐2p states (**Figure** **5**a). Since the electronic leaps near the Fermi level determine the optical properties of compounds, therefore, the optical properties of C_4_N_3_H_6_SO_3_NH_2_ mainly depend on the π‐conjugated [C_4_N_3_H_6_] units. The electron density difference (EDD) was also calculated. As shown in Figure 5d, the π‐electron cloud is uniformly distributed around the [C_4_N_3_H_6_] group, clearly demonstrating that the π‐conjugated group [C_4_N_3_H_6_] determines the optical properties of the title compound. Since C_4_N_3_H_6_SO_3_NH_2_ crystallizes in the monoclinic crystal system, the title compound is a biaxial crystal. Figure 5b shows the refractive indexes have the relation of *n*_y_ > *n*_z_ > *n*_x_. The refractive indexes of C_4_N_3_H_6_SO_3_NH_2_ at *λ* = 546 nm are *n*_x_ = 1.600, *n*_y_ = 1.820 and *n*_z_ = 1.788, respectively (Figure 5c), so the theoretical birefringence index is 0.220 @ 546 nm, which fits well with experimental birefringence. According to Kleinman symmetry,^[^ ^31^ ^]^ the *m*‐point group of C_4_N_3_H_6_SO_3_NH_2_ leads to six independent NLO coefficients: *d*_11_, *d*_12_, *d*_13_, *d*_15_, *d*_24_, and *d*_33_. The calculated SHG tensors are *d*_11_ = 0.11 pm V^−1^, *d*_12_ = 1.64 pm V^−1^, *d*_13_ = 0.36 pm V^−1^, *d*_15_ = 0.23 pm V^−1^, *d*_24_ = 0.033 pm V^−1^, and *d*_33_ = 1.25 pm V^−1^. The largest SHG tensor *d*_12_ is approximately 4.2 times that of KDP (*d*_36_ = 0.39 pm V^−1^), which is basically in agreement with the experimental result. The origin of the SHG response was also calculated using the SHG density method.^[^ ^32^ ^]^ The SHG density can be divided into occupied states of virtual‐electron (VE) and unoccupied states of virtual‐hole (VH), respectively. For C_4_N_3_H_6_SO_3_NH_2_, the VE process was analyzed due to its major contribution to the SHG response. From Figure 5e,f, it can be seen that the N2, N3, C1, and C2 atoms are the main contributors to the microscopic second‐order NLO response of the occupied state. Thus, the SHG response of the crystals is mainly contributed by the planar π‐conjugated group [C_4_N_3_H_6_].

## 3 Conclusion

In summary, a multifunctional organic–inorganic hybrid crystal C_4_N_3_H_6_SO_3_NH_2_ has been synthesized using an aqueous solution method. In the crystal structure, the [NH_2_SO_3_] group connects the [C_4_N_3_H_6_] layer via hydrogen bonds to form a 3D network structure. The Hirshfeld surface analysis further indicates that the intermolecular interaction is dominated by strong hydrogen bonding between organic and inorganic motifs, leading to coplanar configuration of [C_4_N_3_H_6_] groups. The title compound exhibited strong SHG response (2.5 × KDP) and large birefringence (0.233@546 nm). Analyzing the electronic structure based on first principles, both the strong SHG response and the large optical anisotropy originate from the planar π‐conjugated [C_4_N_3_H_6_] group. Also, the emission colors of C_4_N_3_H_6_SO_3_NH_2_ can be switched from blue‐violet to blue‐green depending on the change in UV light illumination from 302 to 365 nm. Further, the flexible transparent fluorescent films by embedding C_4_N_3_H_6_SO_3_NH_2_ into PVA were prepared, showing a UV light‐switchable feature. This work not only enriches the methodology of synthesizing new NLO crystals by organic–inorganic hybridization but also demonstrates the prospect of its application as an optical material with multifunctionality.

## Conflict of Interest

The authors declare no conflict of interest.

## Supporting information

Supporting Information

## Data Availability

The data that support the findings of this study are available from the corresponding author upon reasonable request.
