# Supplementary material for: Rational Combination of π‐Conjugated and Non‐π‐Conjugated Groups Achieving Strong Nonlinear Optical Response, Large Optical Anisotropy, and UV Light‐Switchable Fluorescence
Source: Adv Sci (Weinh). 2024 Mar 13;11(19):2401325. doi: 10.1002/advs.202401325 (PMC11109661; doi:10.1002/advs.202401325)
Supplement: Supplementary file 1 — Supporting Information [file ADVS-11-2401325-s001.pdf]

ADVANCED SCIENCE
Open Access

Supporting Information

for *Adv. Sci.*, DOI 10.1002/advs.202401325

Rational Combination of π-Conjugated and Non-π-Conjugated Groups Achieving Strong Nonlinear Optical Response, Large Optical Anisotropy, and UV Light-Switchable Fluorescence

Danyang Dou, Qi Shi, Huimin Li, Bingbing Zhang, Daqing Yang and Ying Wang**

SUPPLEMENTARY INFORMATION

Rational Combination of π-conjugated and Non-π-conjugated Groups Achieving Strong Nonlinear Optical Response, Large Optical Anisotropy, and UV Light-Switchable Fluorescence

Danyang Dou,[a] Qi Shi,[a] Huimin Li,[a] Bingbing Zhang,[ab] Daqing Yang,[a]* and Ying Wang*[ab]

[a] *Hebei Research Center of the Basic Discipline of Synthetic Chemistry, Key Laboratory of Analytical Science and Technology of Hebei Province, College of Chemistry and Materials Science, Hebei University, Baoding 071002, China.*

[b] *Institute of Life Science and Green Development, Hebei University, Baoding 071002, China. Email: wangy@hbu.edu.cn.*

Table of Contents

Experimental section S2

Table S1. Crystallographic information for $C_4N_3H_6SO_3NH_2$. S4

Table S2. Atomic coordinates and U_{eq} [Å^2] for $C_4N_3H_6SO_3NH_2$. S5

Table S3. Bond lengths [Å] and angles [°] for $C_4N_3H_6SO_3NH_2$. S6

Table S4. Hydrogen bonds for $C_4N_3H_6SO_3NH_2$. S7

Figure S1. 2D Fingerprint plots for (a) overall interactions and (b–i) individual interactions of atom types in crystal packing of $C_4N_3H_6SO_3NH_2$. S8

Figure S2. SEM images and EDX microanalysis for $C_4N_3H_6SO_3NH_2$. S9

Figure S3. The IR spectrum for $C_4N_3H_6SO_3NH_2$. S10

Figure S4. CIE color chromaticity of $C_4N_3H_6SO_3NH_2$ phosphors under the excitation of (a) 342nm and (b) 398nm. S11

Figure S5. Calculated band structure for $C_4N_3H_6SO_3NH_2$. S12

Reference S13

Experimental section.

Crystal synthesis. Single crystals of $C_4N_3H_7SO_3NH_2$ were prepared by evaporation of aqueous solution. 2-Aminopyrimidine and HNH_2SO_3 were mixed in a 1:1 ratio in 5 ml of deionised water. The mixed solution was thoroughly stirred and placed in an oven at 40 °C to slowly evaporate, and colourless solid crystals were obtained after one week.

Luminescent thin films preparation. An aqueous solution of $C_4N_3H_6SO_3NH_2$ with a concentration of 10 mg/ml was prepared by dissolving 0.1 g of $C_4N_3H_6SO_3NH_2$ powder in 10 ml of deionized water. A PVA solution with a mass fraction of 5 % was prepared by weighing 1 g of PVA and dissolving it in 19 ml of deionized water. 0.2 ml of $C_4N_3H_6SO_3NH_2$ aqueous solution was mixed with 10 ml of PVA solution to obtain the complex solution. The resulting solution was slowly added drop by drop to the slide surface and dried at room temperature for 24 hours. Complete and separate composite films were collected from the surface of the slides.

Single-Crystal X-ray Diffraction. A micron-level colorless transparent single crystal with good quality was selected under the polarizing microscope, and the data were collected by the Bruker D8 VENTURE diffractometer (Mo-Kα radiation). The SAINT[1] and SADABS[2] programs were used for data integration and absorption correction, and the ShelXT and ShelXL[3] were used to process single crystal data. Then, the PLATON[4] program was used to verify the symmetry of the crystal structure. In general, the position of hydrogen atoms could not accurately determined from XRD. However, they can be figure out if high-quality and high-resolution XRD data were acquired. In this work, H atoms were determined by two ways: (1) The position of H atoms (H3, H4, and H5) from $[C_4N_3H_6]$ groups were handled with AFIX 43 command (suitable for riding model refinement), which means an idealized terminal NH_2 with the hydrogen atoms in the plane of the nearest substituent on the N atom. (2) Hydrogen atoms of the amino group in the $[SO_3NH_2]$ group and the $[C_4N_3H_6]$ group, however, have been detected in the experimental electron density in difference Fourier map. A close examination of the residual electron density could locate two possible H positions near the N atom, and "DFIX 0.86 N1 H1" and "DFIX 0.9 N3 H2" commands were used to restraint the two N—H distances. The crystal data and structure refinement for $C_4N_3H_6SO_3NH_2$ are displayed in Table S1. Crystallographic information such as atomic coordinates, bond lengths and angles, and hydrogen bonds for $C_4N_3H_6SO_3NH_2$ are listed in Tables S2-S4.

Powder XRD. The phase purity of the grown crystals was checked by the powder XRD measurement. Dandong Haoyuan DX-27mini X-ray diffractometer (Cu Kα, λ = 1.5406 Å) was used to collect the XRD data at room temperature. The 2θ range was 10-70°, the scanning step was 0.02°, and the scanning time was 2 s.

Scanning electronic microscope (SEM) images and energy dispersive X-Ray (EDX) microanalysis. SEM images and EDX microanalysis of the crystal were

performed on a Hitachi TM4000Plus microscope with an acceleration voltage of 15 kV. The elemental mapping images show the distribution of elements in the crystal.

Thermal analysis. Thermogravimetric analysis (TGA) and differential scanning calorimetry (DSC) were gathered using a NETZSCH STA449F3 thermal analyzer. The crystal samples were heated from room temperature to 800 °C at a rate of 10 °C min^{-1} under air condition.

Infrared (IR) spectrum. The IR spectrum of $C_4N_3H_6SO_3NH_2$ at room temperature was scanned with a Nicolet iS10 spectrometer in the wavelength of 400-4000 cm^{-1}. The crystal sample and KBr were grounded and pressed into a transparent sheet at a ratio of 1:100.

UV-Vis–NIR diffuse reflectance spectrum. The UV-Vis-NIR diffuse reflectance spectrum of $C_4N_3H_6SO_3NH_2$ was collected in a Shimadzu UV-2600i spectrometer in the wavelength range of 200-1100 nm.

Fluorescence spectrometer. The excitation and emission fluorescence spectra of $C_4N_3H_6SO_3NH_2$ were analyzed using a fluorescence spectrometer (FS5, Edinburgh, UK).

Birefringence Measurements. The birefringence was measured by interference colour method [5]using the polarizing microscope (ZEISS Axio Scope. A1) equipped with a quartz wedge compensator at 546 nm. The birefringence index was calculated by the formula $R = \Delta n \times d$, where R, Δn and d were the optical path difference measured by polarizing microscope, the birefringence index and the crystal thickness measured by single-crystal X-ray diffraction, respectively.

Second harmonic generation (SHG) measurement. Under 1064 nm coherent light radiation generated by a Q-switched Nd: YAG laser, the powder SHG effects test was performed by using the modified Kurtz and Perry method.[6] After grinding and sieving, the polycrystalline powder samples were divided into different particle sizes: 20-38.5, 38.5-55, 55-80, 80-125, 125-160, and 160-200 μm. KH_2PO_4 (KDP) samples with the same particle size range were used as the standard.

Theoretical calculation. The CASTEP[7] package was used for theoretical calculation based on density functional theory (DFT).[8] The generalized gradient approximation (GGA) of Perdew-Burke-Ernzerhof (PBE)[9] and HSE06[10] functionals were used to describe the exchange-correlation energy. The basic cutoff energy of a plane wave was identified as 750 eV. The Monkhorst-Pack scheme with a grid density of 4 × 3 × 4 at point *k* is used for the numerical integration of Brillouin zone for $C_4N_3H_6SO_3NH_2$.

Table S1. Crystallographic information for $C_4N_3H_6SO_3NH_2$.

Compound	$C_4N_3H_6SO_3NH_2$
Temperature (K)	273(2)
Crystal system	Monoclinic
space group (number)	*Cm*(8)
a [Å]	7.532(4)
b [Å]	7.116(3)
c [Å]	7.732(4)
α [°]	90
β [°]	105.353(18)
γ [°]	90
Z	2
Theta range for data collection (deg.)	5.46 to 55.01 (0.77 Å)
Limiting indices	$-9 \leq h \leq 9$; $-9 \leq k \leq 9$; $-10 \leq l \leq 10$.
Reflections collected / unique	3045/962
Completeness to theta	100 %
Data / restraints / parameters	962/17/71
μ (mm^{-1})	0.380
$F(000)$	200
GOF on F^2	1.077
R_1, wR_2 ($I>2\sigma(I)$)[a]	0.0570, 0.1045
R_1, wR_2 (all data)[a]	0.0883, 0.1192
Largest diff. peak and hole ($e \cdot Å^{-3}$)	0.65/-0.39
Flack X parameter	0.2(3)
CCDC number	2309611

Table S2. Atomic coordinates and U_{eq} [Å^2] for $C_4N_3H_6SO_3NH_2$.

**Atom**	***x***	***y***	***z***	***U*(eq)**
S1	0.5622(5)	0.500000	0.2581(5)	0.0380(6)
O2	0.6458(7)	0.6724(7)	0.2185(7)	0.0551(15)
O1	0.5450(10)	0.500000	0.4423(10)	0.060(2)
N1	0.5863(14)	1.000000	0.4373(13)	0.053(2)
H1	0.597(10)	1.113(5)	0.402(10)	0.063
N3	0.3565(11)	0.500000	0.1178(11)	0.0403(19)
H2	0.292(8)	0.601(7)	0.135(8)	0.048
N2	0.4839(9)	0.8328(9)	0.6531(10)	0.0559(18)
H3	0.503251	0.727610	0.606438	0.067
C1	0.5171(12)	1.000000	0.5798(13)	0.037(2)
C2	0.4172(9)	0.8375(10)	0.8067(8)	0.068(2)
H4	0.394533	0.725328	0.858787	0.081
C3	0.3863(15)	1.000000	0.8786(15)	0.080(4)
H5	0.342393	0.999999	0.980258	0.096

U_{eq} is defined as 1/3 of the trace of the orthogonalized U_{ij} tensor.

Table S3. Bond lengths [Å] and angles [°] for $C_4N_3H_6SO_3NH_2$.

**Atom–Atom**	**Length [Å]**	**Atom–Atom–Atom**	**Angle [°]**
S1–O2	1.448(5)	O2–S1–O2	115.8(4)
S1–O2$^{\#1}$	1.448(5)	O2–S1–O1	111.2(3)
S1–O1	1.463(7)	O2–S1–O1	111.2(3)
S1–N3	1.639(8)	O2–S1–N3	104.3(3)
N1–C1	1.338(13)	O2–S1–N3	104.3(3)
N1–H1	0.86(3)	O1–S1–N3	109.4(4)
N1–H1$^{\#2}$	0.86(3)	C1–N1–H1	111(5)
N3–H2	0.90(3)	C1–N1–H1	111(5)
N3–H2$^{\#1}$	0.90(3)	H1–N1–H1	139(10)
N2–C1	1.369(8)	S1–N3–H2	111(4)
N2–C2	1.407(10)	S1–N3–H2	111(4)
N2–H3	0.8600	H2–N3–H2	107(9)
C2–C3	1.3300(16)	C1–N2–C2	118.3(7)
C2–H4	0.9300	C1–N2–H3	120.9
C3–H5	0.9300	C2–N2–H3	120.9
		N1–C1–N2	119.7(5)
		N1–C1–N2	119.7(5)
		N2–C1–N2	120.7(9)
		C3–C2–N2	121.0(9)
		C3–C2–H4	119.5
		N2–C2–H4	119.5
		C2–C3–C2	120.8(12)
		C2–C3–H5	119.6
		C2–C3–H5	119.6

Symmetry transformations used to generate equivalent atoms:
#1: +X, 1-Y, +Z; #2: +X, 2-Y, +Z;

Table S4. Hydrogen bonds for $C_4N_3H_6SO_3NH_2$.

D–H···A [Å]	d(D–H) [Å]	d(H···A) [Å]	∠DHA[°]	d(D···A) [Å]
N1–H1···S1$^{\#1}$	0.86(3)	2.95(3)	3.805(4)	170(7)
N1–H1···O2$^{\#2}$	0.86(3)	2.18(4)	2.983(8)	155(7)
N3–H2···O2$^{\#3}$	0.90(3)	2.15(3)	3.035(7)	171(6)
N2–H3···O1	0.86	2.13	2.979(7)	168.3
C2–H4···N3$^{\#4}$	0.93	2.64	3.514(10)	156.8

Symmetry transformations used to generate equivalent atoms:
#1: +X, 1+Y, +Z; #2: +X, 2-Y, +Z; #3: -0.5+X, 1.5-Y, +Z; #4: +X, +Y, 1+Z;

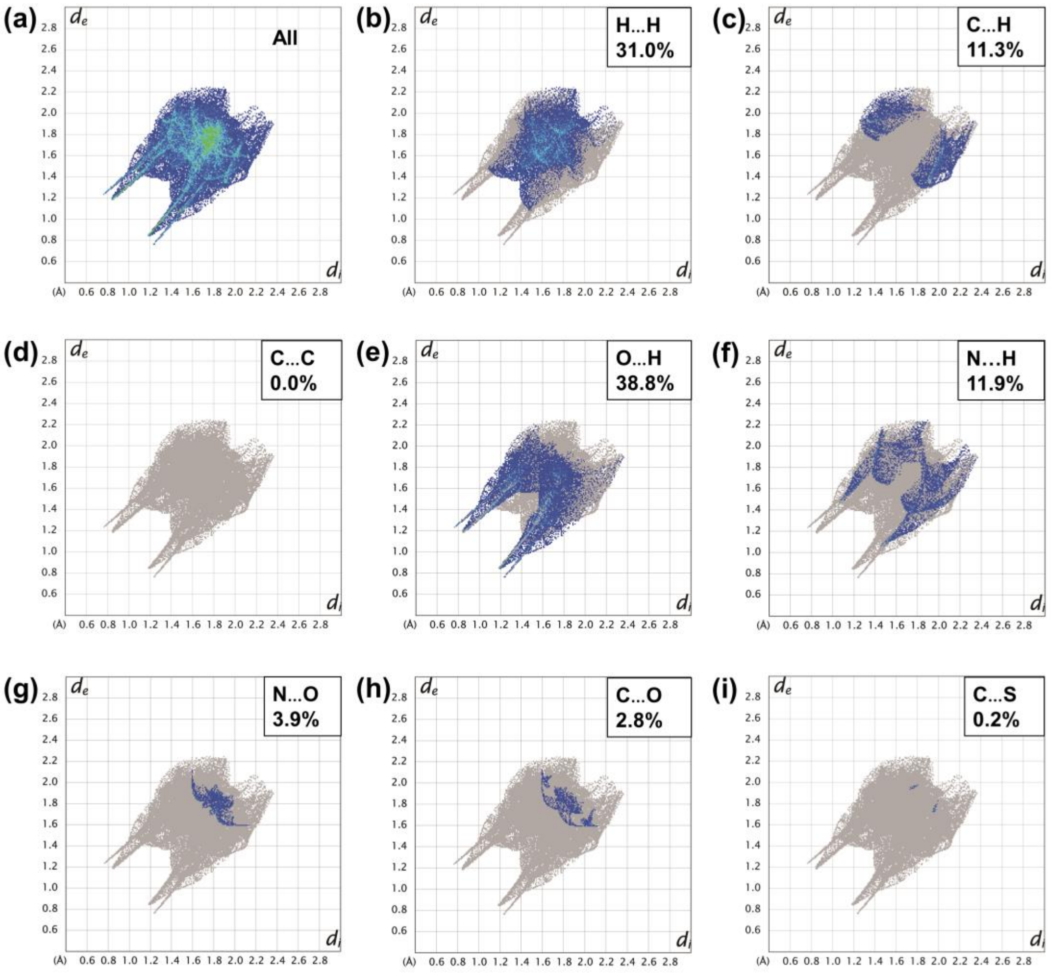

Figure S1. 2D Fingerprint plots for (a) overall interactions and (b−i) individual interactions of atom types in crystal packing of $C_4N_3H_6SO_3NH_2$. Here, d_e and d_i represent distances from the Hirshfeld surfaces to the nearest nucleus outside and inside the surface, respectively.

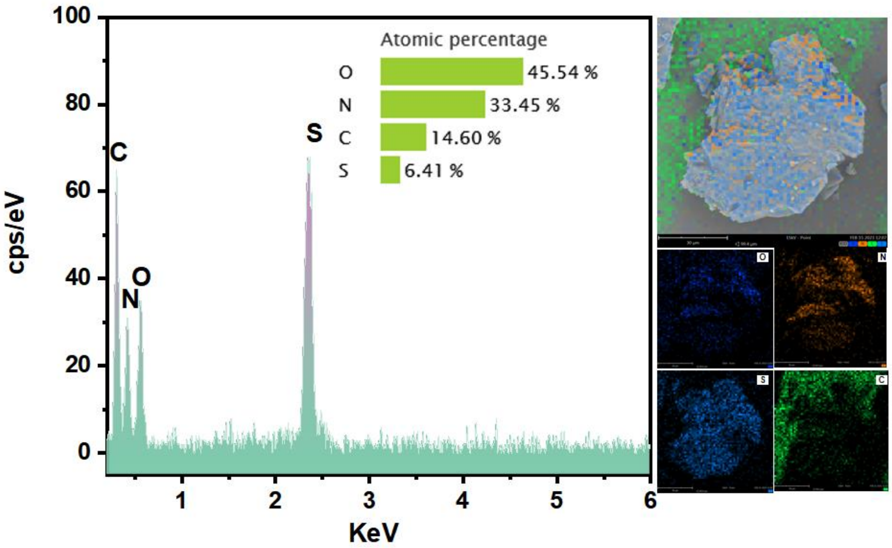

Figure S2. SEM images and EDX microanalysis for $C_4N_3H_6SO_3NH_2$.

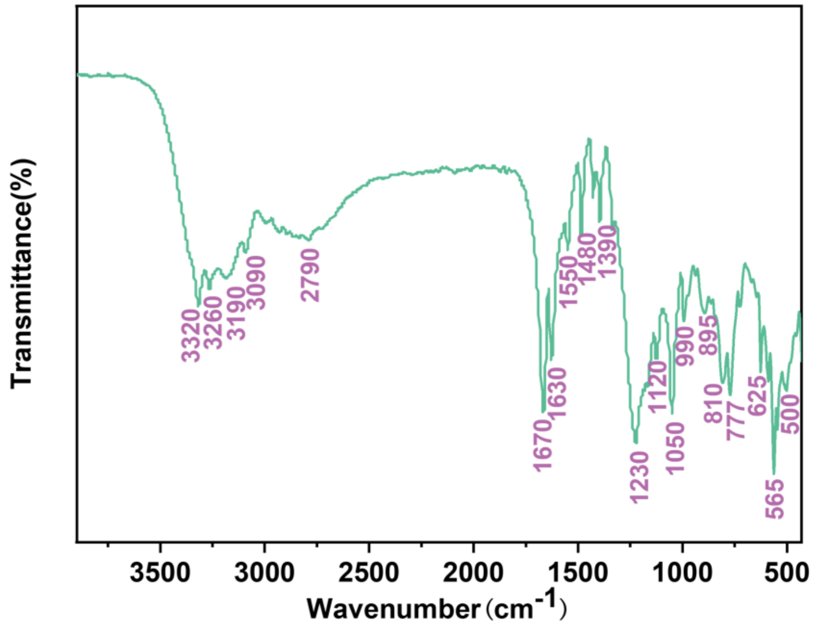

Figure S3. The IR spectrum for $C_4N_3H_6SO_3NH_2$.

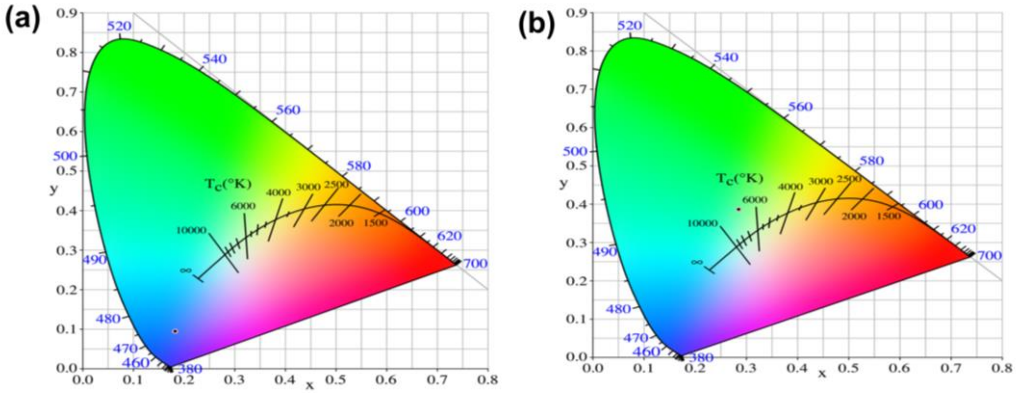

Figure S4. CIE color chromaticity of $C_4N_3H_6SO_3NH_2$ phosphors under the excitation of (a) 342nm and (b) 398nm.

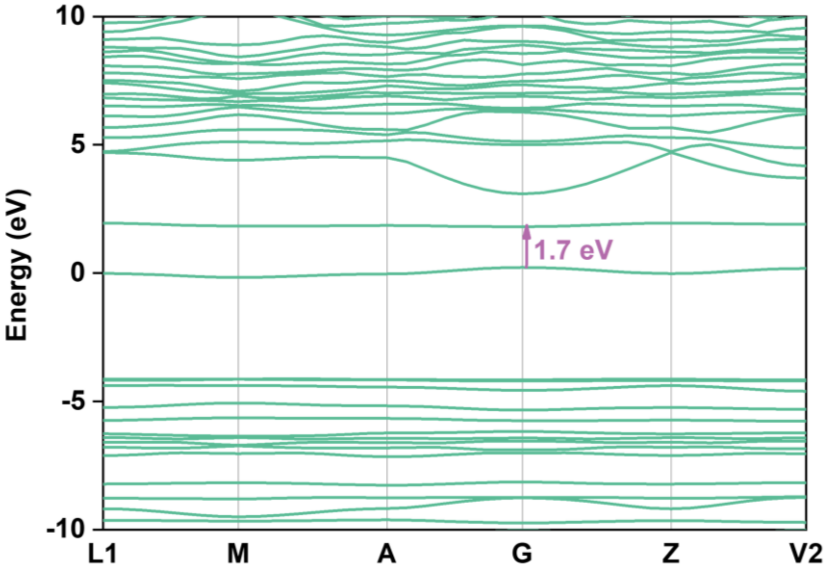

Figure S5. Calculated band structure for $C_4N_3H_6SO_3NH_2$.

Reference.

[1] T. Moriguchi, K. Mitsumoto, Y. Nishizawa, D. Yakeya, V. Jalli, A. Tsuge, *Cryst. Struct. Theory. Appl.* **2016**, *05*, 63-73.

[2] L. Krause, R. Herbst-Irmer, G. M. Sheldrick, D. Stalke, *J. Appl. Cryst.* **2015**, *48*, 3-10.

[3] G. M. Sheldrick, *Acta. Crystallogr. A.* **2008**, *64*, 112-122.

[4] A. L. Spek, *J. Appl. Cryst.* **2002**, *36*, 7-13.

[5] Q. Shi, L. Y. Dong, Y. Wang, *J. Struct. Chem.* **2023**, *42*, 100017.

[6] S. Kurtz, T. Perry, *IEEE J. Quantum Electron.* **1968**, *4*, 333.

[7] S. J. Clark, M. D. Segall, C. J. Pickard, P. J. Hasnip, M. I. J. Probert, K. Refson, M. C. Payne, *Z. Kristallogr. Cryst. Mater.* **2005**, *220*, 567-570.

[8] V. Milman, K. Refson, S. J. Clark, C. J. Pickard, J. R. Yates, S. P. Gao, P. J. Hasnip, M. I. J. Probert, A. Perlov, M. D. Segall, *J. Mol. Struct.* **2010**, *954*, 22-35.

[9] J. P. Perdew, K. Burke, M. Ernzerhof, *Phys. Rev. Lett.* **1996**, *77*, 3865

[10] J. Heyd, G. E. Scuseria, *J. Chem. Phys.* **2004**, *121*, 1187-1192.
